# A randomized double blind placebo controlled clinical trial of N-Acetylcysteine added to risperidone for treating autistic disorders

**DOI:** 10.1186/1471-244X-13-196

**Published:** 2013-07-25

**Authors:** Ahmad Ghanizadeh, Ebrahim Moghimi-Sarani

**Affiliations:** 1 Research Center for Psychiatry and Behavioral Sciences, Shiraz University of Medical Sciences, School of Medicine, Shiraz, Iran; 2 Department of Psychiatry, Shiraz University of Medical Sciences, School of Medicine, Shiraz, Iran

**Keywords:** Autism, Clinical trial, Randomized, Therapy, N-acetylcysteine, Oxidative stress

## Abstract

**Background:**

This study examined the efficacy and safety of N-acetylcysteine (NAC) augmentation for treating irritability in children and adolescents with autism spectrum disorders (ASD).

**Method:**

Forty children and adolescents met diagnostic criteria for ASD according to DSM-IV. They were randomly allocated into one of the two groups of NAC (1200 mg/day)+risperidone or placebo+risperidone. NAC and placebo were administered in the form of effervescent and in two divided doses for 8 weeks. Irritability subscale score of Aberrant Behavior Checklist (ABC) was considered as the main outcome measure. Adverse effects were also checked.

**Results:**

The mean score of irritability in the NAC+risperidone and placebo+risperidone groups at baseline was 13.2(5.3) and 16.7(7.8), respectively. The scores after 8 weeks were 9.7(4.1) and 15.1(7.8), respectively. Repeated measures of ANOVA showed that there was a significant difference between the two groups after 8 weeks. The most common adverse effects in the NAC+risperidone group were constipation (16.1%), increased appetite (16.1%), fatigue (12.9%), nervousness (12.9%), and daytime drowsiness (12.9%). There was no fatal adverse effect.

**Conclusions:**

Risperidone plus NAC more than risperidone plus placebo decreased irritability in children and adolescents with ASD. Meanwhile, it did not change the core symptoms of autism. Adverse effects were not common and NAC was generally tolerated well.

**Trial registration:**

This trial was registered at http://www.irct.ir. The registration number of this trial was IRCT201106103930N6

## Background

Autism spectrum disorders (ASD) are characterized by the three main symptoms of: a) significant impairments in social relationships, b) language and communication deficits, and c) restricted interests. Although, autistic disorders are not very common, their current rates are higher than that of the previously reported rates [1]. About 1.9% of school aged children obtain screening cutoff score for probable autistic disorder [2]. The rate for typical autism in five-year-old children is 6.26 per 10,000 [3]. In addition, the global prevalence of autism spectrum disorders is 62/10 000 [4].

The neurobiology and etiology of autism are not clearly known [5]. However, genetic [6], neurologic, metabolic, and immunologic factors are suggested to be involved. It is proposed that there is an imbalance of oxidative stress and anti-oxidative defenses in children with autism [7,8]. The deficit in antioxidant system is specific in autism [9] and it mediates the association of some behavioral symptoms and immunity function [10]. While plasma antioxidant capacity is decreased [11], the plasma oxidative stress indicators, such as nitric oxide (NO) and malondialdehyde (MDA), are increased [12]. In addition, lipid peroxidation is increased in autism [13]. Oxidative stress markers in urine may represent oxidative stress index in autistic patients and some of them are suggested as the biomarkers of autism [14]. The levels of superoxide dismutase (SOD) and glutathione peroxidase, as antioxidant enzymes, are lower in autism than that of the controls [15].

Oxidative stress negatively affects mitochondrion through respiratory chain [16]. Therefore, oxidative stress is suggested as a target for treating autism [7,17]. In addition, animal models of autism revealed that targeting oxidative markers was effective for treating autism [18].

Glutathione plays a significant role in defense against oxidative stress in autism [7,19]. The level of glutathione in the cerebellum and temporal cortex of patients with autism are markedly decreased (34.2% and 44.6%, respectively) [20]. The levels of both reduced glutathione and total glutathione are lowered in autistic patients than that of the controls [21]. Moreover, glutathione pathway gene variation increases the risk of autistic disorders [22].

Glutathione, which is the most important intracellular defense against oxidative stress, consists of glutamate, glycine, and cysteine [7]. The pathways of methionine cycle, transsulfuration pathway, and GSH-synthesis pathway produce glutathione [23]. The role of cysteine for the production of glutathione is very important because cysteine has a rate-limiting role [7]. Recently, an eight-week, open-label trial showed that glutathione supplementation increased the reduced-form of glutathione in plasma in children with autism spectrum disorders [24]. This supplementation also increased the plasma levels of sulfate, cysteine, and taurine [24].

In addition, glutamate is involved in the pathophysiology of autism [25] and glutamate blockers improve the animal models of autism [26,27]. Some other interventions targeting glutamate are suggested for treating autism as well [28-30].

N-acetylcysteine (NAC) is an antioxidant precursor to glutathione [31]. NAC restores GSH level [31]. In addition, NAC scavenges oxidants such as hydroxyl radical and H_2_O_2_[32]. NAC improves glutamate homeostasis and decreases the relapse rate in drug-dependent patients [33]. NAC is an effective and safe agent for treating schizophrenia [34], bipolar disorder [35], Alzheimer’s disease [36], and nail biting [37]. Moreover, the adverse effects of NAC are mild and self-limited in children [38]. NAC is an over-the-counter supplement.

About 75.5% of patients with autism respond to risperidone while the rate for placebo is 11.5% (effect size=1.2) [39]. Aripiprazole with the effect size of 0.87 is effective too [40]. This trial investigates the efficacy and safety of NAC as an adjuvant treatment with risperidone for treating the irritability of children with ASD. Considering the role of glutathione in autism and the effect of NAC on glutathione level, it is hypothesized that NAC as an augmentation agent decreases irritability score in children with ASD.

## Methods

This study was an eight week randomized double-blind placebo-controlled clinical trial with two parallel groups. The patients, parents, and independent assessor were blind to the allocation of patients. The patients were randomly allocated into one of the two groups using a random number generator.

The participants were a convenient sample of outpatients children aged between 3.5 to 16 years old from both genders. The sample was recruited from the Child and Adolescent Psychiatriy Clinics affiliated with Shiraz University of Medical Sciences, Iran.

The diagnosis of autism was made using DSM-IV-TR criteria. The diagnosis was in accordance with Autism Diagnostic Interview-Revised (ADI-R) [41]. All the interviews were conducted by an expert child and adolescent psychiatrist (A.G.). Assessments were performed by a resident of psychiatry trained to use the questionnaire and checklist. The level of intelligence was not considered as an exclusion criterion. However, the participants should be able to take the medications.

The patients were free from any concomitant medication. Otherwise, the dose of medication should not be markedly changed during two weeks prior entering into this study. The dose of concomitant medication was not markedly changed during this trial.

Patients with psychotic disorders, active substance abuse or dependence, unstable medical condition, evidence of active liver disease, seizure disorder, unstable hypertension or cardiac disease, unstable asthma, and kidney disease as determined by the investigator were not included. Taking concomitant medications with glutamatergic effects (e.g., dextromethorphan, D-cycloserine, amantadine, memantine, lamotrigine, riluzole) was not allowed. In addition, those with hypersensitivity/allergy to NAC were not included.

One of the two groups received risperidone plus N-Acetylcysteine (1200 mg/day). The patients received 1200 mg/day NAC in two divided doses. Both NAC and placebo tablets were administered in the form of effervescent. The other group received risperidone plus placebo tablets. The shape, size, taste, and color of NAC and placebo were identical.

The dose was titrated up during two weeks. The dose was not fixed. In addition, all the patients in both groups received risperidone. Risperidone started at the dose of 0.5 mg/day and it was titrated up to 2 mg/day during three weeks for children less than 30 kg. The dose for children more than 30 kg was up to 3 mg/day [42,43]. The dose of risperidone was not fixed and it could be changed considering the clinical symptoms and adverse effects.

The primary outcome measure was Aberrant Behavior Checklist [44]. ABC consists of 58 items and includes 5 subscales. The subscales are Irritability, Lethargy and Social Withdrawal, Stereotypic Behavior, Hyperactivity and Noncompliance, and Inappropriate Speech.

The changes of Irritability subscale score was considered as the main outcome of the current trial.

It was completed at pre-intervention, 4 weeks after the beginning of intervention, and 8 weeks after the beginning of intervention. In addition, the adverse effects and extrapyramidal symptoms were systematically examined and assessed using checklists [43]. The patients and their parents were also asked regarding possible adverse effects.

This trial’s protocol and procedures were approved by Ethics Committee of Shiraz University of Medical Sciences, Iran. Then, this trial was registered at http://www.irct.ir. The registration number of this trial was IRCT201106103930N6. This trial was performed in 2011 and 2012. The parents of children provided written informed consent.

### Analysis

SPSS was used to perform statistical analyses. Chi-Square test was used to compare the gender ratio between the two groups. The mean of age was compared between the two groups using independent t-test. Repeated Measures of ANOVA was used to examine the effect of interventions on the subscales scores of ABC. Intent-to-Treat (ITT) using Last Observed Carried Forward (LOCF) with at least one post-treatment evaluation was used to handle missing data [45]. The Cohen’s d was calculated to measure effect size. P value less than 0.05 was set for being statistically significant. This is an exploratory small sample size trial.

## Results

**Figure 1:**
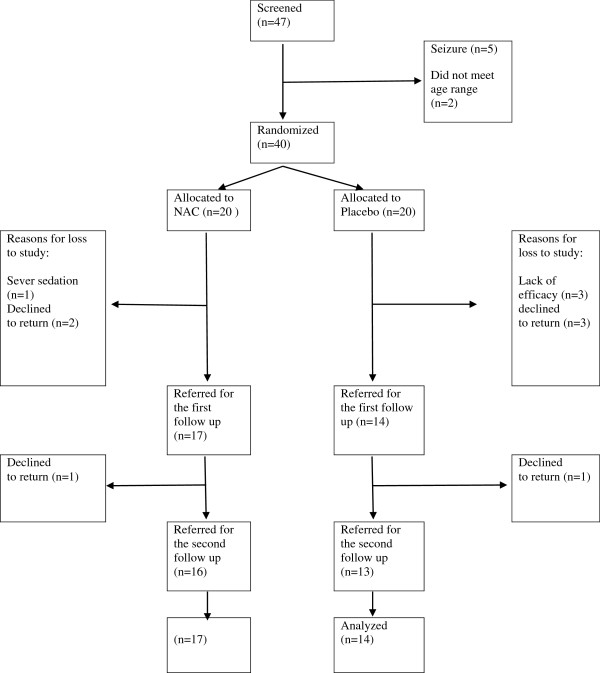
Flow chart for the clinical trial N-Acetylcysteine + risperidone versus Placebo+risperidone.

Out of 47 children who were screened, 40 patients were randomized into one of two groups. The reasons for the drop of patients in the two groups are displayed in Figure 1. The number of boys in the NAC and placebo groups was 13 and 12, respectively. There was no statistically significant difference between the two groups regarding gender ratio (X2=.4, df=1, P=0.5). In addition, the mean of age was not different between the two groups (t=0.4, df=29, P=0.4). The mean age of children in the NAC and placebo group was 8.8(3.1) and 7.9(2.4) years, respectively. The list of concomitant medications is presented in Table 1. The mean (SD) dose of risperidone in the NAC+risperidone and placebo+risperidone groups was 0.76(0.2) mg/day and 0.92(0.3) mg/day, respectively. It was not significantly different between the two groups (t=1.4, df=29, P=0.1).

**Table 1:** The number of patients taking concomitant medications during this trial

**Medication (Mean dose)**	**N-Acetylcysteine+risperidone group (Number)**	**Placebo+risperidone group (Number)**
Clonidine (0.75 mg/day)	5	3
Folic acid (1 mg/day)	2	0
Imipramine (10 mg/day)	1	0
Biperiden (2 mg/day)	1	1
Nortriptyline (12.5 mg/day)	1	0
Topiramate (12.5 mg/day)	0	2

**Figure 2:**
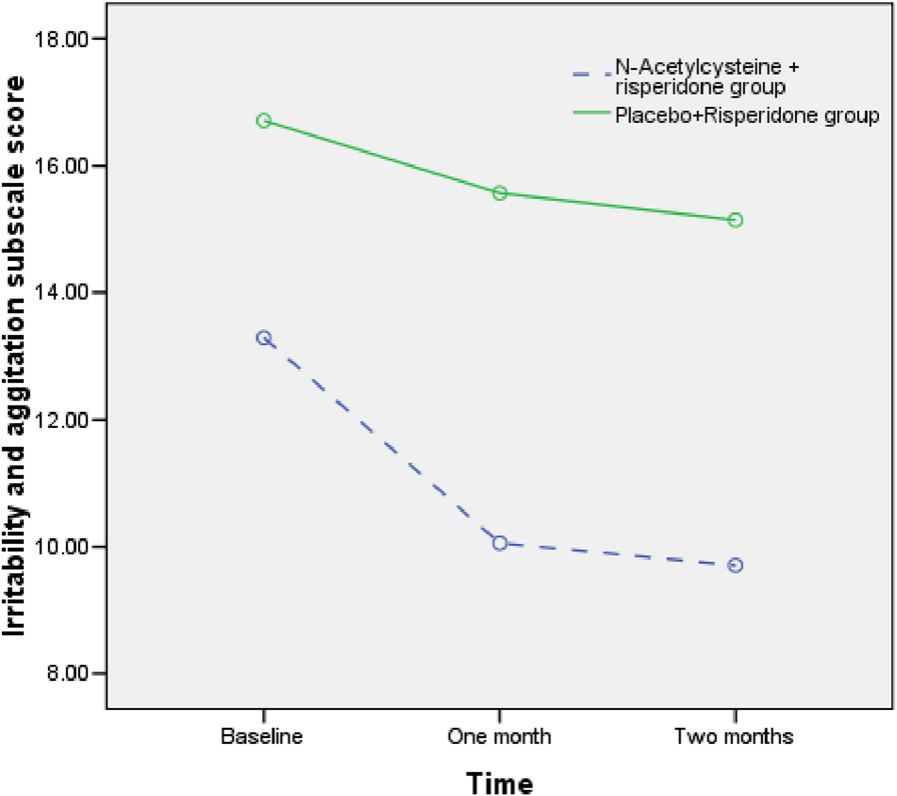
Comparison of irritability subscale scores between N-Acetylcysteine + risperidone group and Placebo+Risperidone group.

Repeated measures ANOVA showed both NAC and Placebo decreased Irritability subscale score during the trial (F1.4,43.0=10.5, P<0.001) (Figure 2). The irritability scale score decreased from 13.2(5.3) to 9.7(4.1) in the NAC+risperidone group. However, the irritability score decreased 16.7(7.8) to 15.1(7.8) in the placebo+risperidone group. Repeated measures ANOVA showed that the effects of groups were significantly different (F1,29=4.9, P<0.035). Moreover, the interactive effect of time and group (time×group) was not statistically different (Table 2). The pattern of changes for Irritability subscale score was different between the NAC+risperidone and placebo+risperidone groups. NAC+risperidone more than placebo+risperidone decreased this score (effect size= .14). There was no difference between the two groups regarding Lethargy and Social Withdrawal, Stereotypic Behavior, Hyperactivity and Noncompliance, and Inappropriate Speech subscales scores.

**Table 2:** The mean (standard deviation) scores of ABC subscales in the N-Acetylcysteine + risperidone group and Placebo+risperidone group

**Subscale**	**Group**	**Baseline score**	**One month after the onset of intervention**	**Two months after the onset of intervention**	**Between groups difference**
Irritability	N-Acetylcysteine + risperidone	13.2(5.3)	10.0(4.1)	9.7(4.1)	F1,29= 4.9, P<0.035
	Placebo+Risperidone	16.7(7.8)	15.5(7.9)	15.1(7.8)	
Hyperactivity and Noncompliance	N-Acetylcysteine + risperidone	29.3(6.4)	21.4(7.7)	18.3(6.9)	F1,29= 2.4, P=0.12
	Placebo+Risperidone	31.9(8.9)	26.8(11.0)	24.3(12.1)	
Lethargy and Social Withdrawal	N-Acetylcysteine + risperidone	11.9(6.5)	9.0(5.7)	8.5(6.5)	F1,28= 0.4, P=0.53
	Placebo+Risperidone	12.2(8.3)	11.0(7.8)	10.9(7.5)6	
Stereotypic Behavior	N-Acetylcysteine + risperidone	6.6(4.5)	4.5(3.6)	3.9(2.7)	F1,28= 2.8, P=0.1
	Placebo+Risperidone	8.5(6.3)	7.7(6.2)	7.8(6.6)	
Inappropriate Speech	N-Acetylcysteine + risperidone	3.9(3.7)	3.8(3.7)	3.2(3.4)	F1,29= 1.8, P=0.1
	Placebo+Risperidone	5.7(3.8)	5.3(3.7)	5.2(4.0)	

### Adverse effects

Adverse effects were also checked. None of the participants experienced any fatal adverse effect. Only one patient withdrew due to adverse effect in the NAC group. The adverse effects were usually mild. The most common adverse effects were constipation (16.1%), increased appetite (16.1%), fatigue (12.9%), nervousness (12.9%), and daytime drowsiness (12.9%) (Table 3).

**Table 3:** The number of patients with the adverse effects in the N-Acetylcysteine + risperidone group and Placebo+risperidone group

**Adverse effect**	**N-Acetylcysteine + risperidone group**		**Placebo+risperidone group**	
	**Number**	**Percent**	**Number**	**Percent**
Daytime drowsiness	4	12.9	2	6.5
Morning drowsiness	1	3.2	0	0
Stiffness	2	6.5	0	0
Slowed movement	2	6.5	0	0
Decreased Appetite	1	3.2	-	0
Fatigue	4	12.9	4	3.2
Constipation	5	16.1	1	3.2
Increased appetite	5	16.1	3	9.7
Decreased appetite	2	6.5	1	3.2
Diarrhea	0	0	1	3.2
Tremor	2	6.5	0	0
Abdominal pain	1	3.2	0	0
Nervousness	4	12.9	0	0
Itches	1	3.2	1	3.2
Restlessness	2	6.5	2	6.5
Twitches	1	3.2	0	0
Blurred vision	1	3.2	0	0
Urinary retention	0	0	0	0
Dizziness	0	0	0	0
Skin rash	0	0	0	0
Itches	0	0	0	0
Dry mouth	0	0	0	0
Swallowing difficulty	0	0	0	0
Seizure	0	0	0	0

## Discussion

This randomized double-blind placebo-controlled clinical trial showed that NAC as an adjuvant therapy more than placebo decreased Irritability score in children with ASD. This result suggests that oral NAC as a supplement may be used in order to decrease the level of irritability in children with autistic disorders. Considering the low level of glutathione in autism [7], it is possible that NAC may increase the production of glutathione and enhance anti-oxidative stress system. Our results are in similar line to a recently published case report [46] and a pilot trial showed that NAC more than placebo decreased irritability in children with autism [47]. In addition, our results are very similar to a previously published study reported that NAC was tolerated well and there was no serious adverse effect [47]. The study by Hardan et al., is the only study reported that oral NAC is effective for treating autism [47]. Their participants were taking constant concomitant psychotropic medications and behavioral interventions. However, the type and dosage of the psychotropic medications were not reported. The most common adverse effects of the previous study were constipation, nausea, and diarrhea [47].

### Limitation and future direction

The sample size was small and the duration of current trial was very short. It is not clear whether the symptom will relapse after the discontinuation of NAC. In addition, it is not examined whether this effect will be stable in long term. Further studies may investigate whether NAC enhances anti-oxidative stress system or NAC decreases hyperglutaminergic state in ASD. In order to have a more homogenous group, further studies should not include a wide range of children. In addition, it is recommended to assess the quality of life using other secondary measures in further studies. Finally, the concomitant use of risperidone may limit the efficacy of obtained results. Therefore, the efficacy of higher doses of NAC should be investigated in further trials.

## Conclusion

NAC+risperidone decreased irritability in this trial. This improvement in the NAC+risperidone group was more than that of the placebo+risperidone group. However, NAC no more than placebo decreased other ABC subscales scores including: Lethargy and Social Withdrawal, Stereotypic Behavior, Hyperactivity and Noncompliance, and Inappropriate Speech. Future trials with larger sample sizes, longer durations, and higher doses are required in order to examine the possible effects of NAC on autism.

## Competing interests

The authors declare that they have no competing interests.

## Authors’ contributions

AG was responsible for the initial conception and drafting of the manuscript. AG and EM gathered the data and revised the preliminary draft of the manuscript. Both authors read and approved the final manuscript.

## Pre-publication history

The pre-publication history for this paper can be accessed here:

http://www.biomedcentral.com/1471-244X/13/196/prepub
